# Evaluation of Temozolomide Treatment for Glioblastoma Using Amide Proton Transfer Imaging and Diffusion MRI

**DOI:** 10.3390/cancers14081907

**Published:** 2022-04-10

**Authors:** Ryutarou Onishi, Reika Sawaya, Keiho Tsuji, Narumi Arihara, Akiko Ohki, Junpei Ueda, Junichi Hata, Shigeyoshi Saito

**Affiliations:** 1 Department of Medical Physics and Engineering, Area of Medical Imaging Technology and Science, Division of Health Sciences, Osaka University Graduate School of Medicine, Osaka 565-0871, Japan; u292923c@ecs.osaka-u.ac.jp (R.O.); u010443b@ecs.osaka-u.ac.jp (R.S.); u768334g@ecs.osaka-u.ac.jp (K.T.); u478276a@ecs.osaka-u.ac.jp (N.A.); okiakiko@kuhp.kyoto-u.ac.jp (A.O.); uedaj@sahs.med.osaka-u.ac.jp (J.U.); 2 Division of Clinical Radiology Service, Kyoto University Hospital, Kyoto 606-8507, Japan; 3 Graduate School of Human Health Science, Tokyo Metropolitan University, Tokyo 116-8551, Japan; j-hata@tmu.ac.jp; 4 Department of Advanced Medical Technologies, National Cerebral and Cardiovascular Center Research Institute, Osaka 564-8565, Japan

**Keywords:** glioblastoma, chemotherapy with temozolomide, 7T-MRI, amide proton transfer imaging, neurite orientation dispersion and density imaging

## Abstract

**Simple Summary:**

Glioblastoma (GBM), the most frequent and malignant histological type of glioma, is associated with a very high mortality rate. MRI is a useful method for the evaluation of tumor growth. However, there are few studies that have quantitatively evaluated the changes in disease state after TMZ treatment against GBM, and it is not fully understood how the effects of treatment are reflected in the quantitative values measured on MRI. We used the C6 glioma rat model to evaluate the tumor changes due to chemotherapy at different doses of TMZ in terms of quantitative values measured by neurite orientation dispersion and density imaging (NODDI) and amide proton transfer (APT) imaging using 7T-MRI. These methods can evaluate the microstructural changes caused by TMZ-induced tumor growth inhibition.

**Abstract:**

This study aimed to evaluate tumor changes due to chemotherapy with temozolomide (TMZ) in terms of quantitative values measured by APT imaging and NODDI. We performed TMZ treatment (administered orally by gavage to the TMZ-40 mg and TMZ-60 mg groups) on 7-week-old male Wistar rats with rat glioma C6 implanted in the right brain. T_2_WI, APT imaging, diffusion tensor imaging (DTI), and NODDI were performed on days 7 and 14 after implantation using 7T-MRI, and the calculated quantitative values were statistically compared. Then, HE staining was performed on brain tissue at day 7 and day 14 for each group to compare the results with the MR images. TMZ treatment inhibited tumor growth and necrotic area formation. The necrotic areas observed upon hematoxylin and eosin (HE) staining were consistent with the MTR low-signal areas observed upon APT imaging. The intracellular volume fraction (ICVF) map of the NODDI could best show the microstructure of the tumor, and its value could significantly highlight the difference in treatment effects at different TMZ doses. APT imaging and NODDI can be used to detect the microstructural changes caused by TMZ-induced tumor growth inhibition. The ICVF may be useful as a parameter for determining the effect of TMZ.

## 1. Introduction

Gliomas are the most common malignant tumors of the central nervous system [1,2]. Glioblastoma (GBM), the most frequent and most malignant histological type of glioma, is associated with a very high mortality rate [3,4]. The global standard of care for GBM is the surgical removal of as much of the tumor as possible, followed by radiotherapy and temozolomide (TMZ) chemotherapy [5,6]. However, there is no completely effective treatment, and the 5-year survival rate is 7.2% [7]. The limiting diagnostic factors include the inability of standard MRI sequences (such as T_2_-weighted imaging (T_2_WI)), fluid-attenuated inversion recovery, and gadolinium T_1_-weighted imaging) to accurately indicate the extent of tumor invasion and tumor grade preoperatively and determine treatment effects [8,9,10].

Advanced diffusion techniques, such as diffusion tensor imaging (DTI), have been widely used for the diagnosis of gliomas [11,12,13]. The fractional anisotropy (FA) value and apparent diffusion coefficient (ADC) value extracted from DTI are used for grading and differentiation between primary and metastatic tumors [12,14,15]. However, these DTI parameters lack specificity in measuring changes in tissue microstructure due to neuropathology [16].

Recently, an advanced diffusion MRI model, neurite orientation dispersion and density imaging (NODDI), has emerged as a powerful tool for evaluating brain microstructure in vivo [17]. NODDI adopts a three-compartment biophysical model, including intracellular, extracellular, and cerebrospinal fluid for each voxel [16]. NODDI can derive the intracellular volume fraction (ICVF), which is a marker of neuronal density, and orientation dispersion index (ODI), which characterizes the angular variation of neurites and the volume fraction of the isotropic compartment (ISO). NODDI can image and quantify these three components separately and is expected to provide new information that reflects changes in the microstructure of the brain better than conventional diffusion MRI [16,18]. Previous studies have reported the usefulness of NODDI in differentiating between primary and metastatic tumors [19,20,21], identifying areas of tumor invasion [22], and grading [23,24,25,26]. There are no papers evaluating the pathological changes in GBM caused by TMZ treatment using NODDI.

Chemical exchange saturation transfer (CEST) imaging is a noninvasive molecular imaging technique that can measure low-concentration endogenous metabolites in humans and animals, such as amide protons [27], glutamate [28], lactate [29,30], and creatine [29,31]. In tumor imaging with MRI, amide proton transfer (APT) imaging, which is the most studied type of CEST imaging, is promising [27,32,33]. APT imaging can selectively detect the increased concentration of amide protons of mobile proteins and peptides in tumors [33]. Magnetization transfer ratio (MTR) values from APT imaging are useful for grading brain tumors [34,35,36,37], predicting isocitrate dehydrogenase (IDH) mutations [38], differentiating treatment-related changes for recurrent tumors [39], and determining early treatment responses [6].

Both NODDI and APT imaging have attracted recent attention as new tools for the diagnosis of brain tumors. Previous studies on brain tumors using the two technologies have much in common in terms of the purpose of their use (such as grading, the identification of the tumor invasive area, differentiating primary and metastatic tumors, and the prediction of IDH mutations). However, there are no studies that have multi-parametrically assessed gliomas using two techniques. In addition, there are few studies that have quantitatively evaluated the changes in disease state after TMZ treatment, and it is not fully understood how the effects of treatment are reflected in the quantitative values measured in NODDI and APT imaging.

In this study, we used the C6 glioma rat model, which is the most widely used GBM model and has high similarity to human GBM, to evaluate tumor changes due to chemotherapy at different doses of TMZ in terms of quantitative values measured by NODDI and CEST imaging (APT) using 7T-MRI.

## 2. Materials and Methods

### 2.1. Cell Culture

C6 rat glioma cells were purchased from the JCRB Cell Bank (National Institutes of Biomedical Innovation, Health and Nutrition, Osaka, Japan). We cultured the cells in Dulbecco’s modified Eagle’s medium supplemented with 10% fetal bovine serum and 1% penicillin–streptomycin solution at 37 °C with 5% CO_2_.

### 2.2. Animal Preparation

All the experimental protocols were approved by the Research Ethics Committee of Osaka University. All the experimental procedures involving animals and their care were carried out in accordance with the Osaka University Guidelines for Animal Experimentation (28-05-1) and the National Institutes of Health Guide for the Care and Use of Laboratory Animals. The animal experiments were performed on 25 7-week-old (170–240 g) male Wistar rats purchased from Japan SLC (Hamamatsu, Japan). All the rats were housed in a controlled vivarium environment (24 °C; 12:12 h light:dark cycle) and fed a standard pellet diet and water ad libitum. A brain tumor model was created by transplanting C6 rat glioma cells directly into the right brain. We first anesthetized the rat with a mixture of air and 4% isoflurane (Wako Pure Chemical Industries, Ltd., Osaka, Japan). During the operation, the rats were immobilized using brain retainers (Stereotaxic Instruments for Rats, NARISHIGESCIENTIFIC INSTRUMENT LAB, Tokyo, Japan) and anesthetized continuously with 2% isoflurane. Second, we used an electric drill to make a hole 4 mm to the right of the bregma. Then, 4.4 × 10^5^/5 μL of C6 cells were injected into the striatum at a depth of 4 mm from the cranial surface at an injection rate of 1.0 μL/min using a micro-syringe.

### 2.3. TMZ Treatment

**Figure 1:**
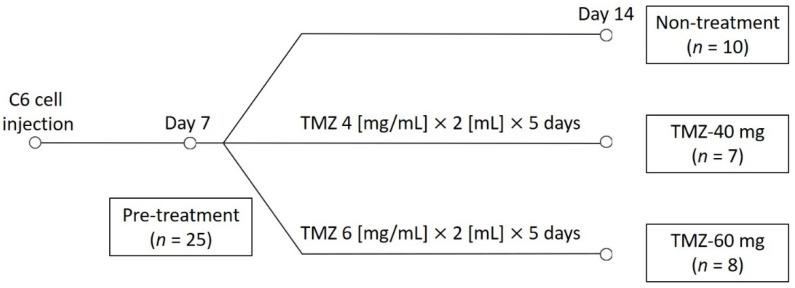
Schedule of the study. TMZ, temozolomide; TMZ-40 mg, the group that received a solution of TMZ dissolved in saline at a concentration of 4 mg/mL orally for 5 consecutive days starting on day 7; TMZ-60 mg, the group that received a solution of TMZ dissolved in saline at a concentration of 6 mg/mL orally for 5 consecutive days starting on day 7.

Rats were administered TMZ (PHR1437; Sigma-Aldrich, St. Louis, MO, USA) solution by gavage. The rats were randomly assigned to three groups: 10 rats were selected for the non-treatment group, 7 for the TMZ-40 mg group, and 8 for the TMZ-60 mg group. TMZ was dissolved in sterile saline at concentrations of 4 mg/mL and 6 mg/mL. The TMZ 4 mg/mL solution and TMZ 6 mg/mL solution were administered orally by gavage to the TMZ-40 mg group and TMZ-60 mg group, respectively, for 5 days starting on day 7 post-injection. The schedule of the study is shown in Figure 1.

### 2.4. Rat MRI

MRI was performed using a 7.0-T MRI, equipped with a transmit/receive volume radio frequency (RF) coil with a diameter of 40 mm (PharmaScan 70/16 US; Bruker BioSpin, Ettlingen, Germany). Axial T_2_WIs were acquired using the turbo rapid acquisition with the relaxation enhancement (Turbo RARE) sequence with the following parameters: repetition time (TR)/echo time (TE) = 3200/33 ms; number of slices = 11; RARE factor = 8; number of averages = 2; field of view = 32.0 × 32.0 mm^2^; matrix size = 128 × 128; slice thickness = 1.0 mm; and scan time = 1 min 42 s.

Two-shell DWIs were acquired using multi-shot echo-planar imaging with the following parameters: TR/TE = 3000/33 ms; number of slices = 11; number of averages = 1; field of view = 19.2 × 19.2 mm^2^; matrix size = 128 × 128; slice thickness = 1 mm; number of shots = 4; fat suppression = on; diffusion directions = 30; b-value shells = 0, 1000, and 2000 s/mm^2^; partial Fourier transform acceleration = 1.5; zero filling factor = 1.3; and scan time = 8 min 40 s.

CEST imaging was performed using rapid acquisition with RARE with the following parameters: TR/TE = 2200/33 ms; number of averages = 1; field of view = 32.0 × 32.0 mm^2^; matrix size = 128 × 128; slice thickness = 1 mm; length = 2000 ms; interpulse delay = 0.01 ms; number of pulses = 20; amplitude = 3.0 μT; pulse shape = block pulse; and scan time = 13 min. Z-spectrum data were acquired from CEST images with varying saturation frequencies from −5.0 ppm to +5.0 ppm in 0.5 ppm steps. S_0_ images (without saturation RF pulses) were acquired before CEST images.

### 2.5. Image Analysis

**Figure 2:**
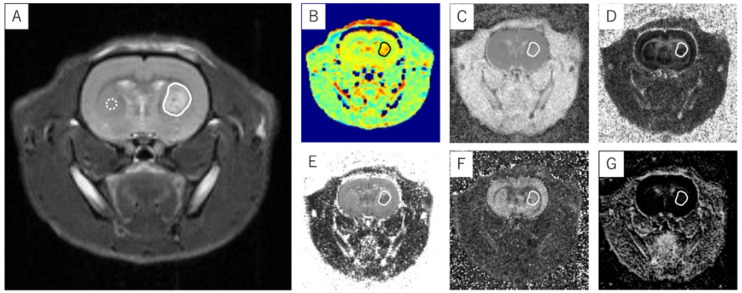
An example of ROI placement in a glioblastoma model. An ROI that covered the entire tumor was placed at the T_2_WI slice position (**A**) where the brain tumor was most widely observed. The ROI was replicated in APT imaging (**B**) and ADC (**C**), FA (**D**), ICVF (**E**), ODI (**F**), and ISO maps (**G**) at the same slice position as the T_2_WI, and each measurement was performed. The T_2_ signal intensity ratio was calculated as the ratio of the average signal value in the solid line ROI to the average signal value in the dotted line ROI. Solid line ROI: transplanted tumor; dotted line ROI: contralateral normal brain tissue.

The FA and ADC were calculated using ParaVision 7 (PharmaScan 70/16 US; Bruker BioSpin, Ettlingen, Germany). The NODDI parameters, including the ICVF, ISO, and ODI, were calculated using MATLAB (MathWorks, Natick, MA, USA) [40,41]. Analyses and calculations for APT imaging were performed using MATLAB (MathWorks) [40,41]. The MTR asymmetry (MTR value) was calculated using the following equation: MTR*_asym_*(%) = (S_[−αppm]_ − S_[+αppm]_)/S_0_. The APT CEST signal was defined as an MTR asymmetry of +3.5 ppm. The MTR asymmetry is expressed as the mean ± standard deviation (SD). All the T_2_WI signal intensity ratios and MTR, ADC, FA, ICVF, ODI, and ISO values were measured using ImageJ 1.53a (National Institutes of Health, Bethesda, MD, USA). The ROIs were placed around the entire tumor excluding hemorrhage and necrosis based on T_2_WI and ADC maps (Figure 2). The tumor volume was defined as the total area of all the T_2_WIs showing the tumor multiplied by the slice thickness of 1.0 mm. The T_2_ signal intensity ratio was calculated as the ratio of the mean signal value of the tumor in the solid ROI to the mean signal value of the contralateral normal brain tissue in the dotted ROI (Figure 2).

### 2.6. Histological Studies

We used hematoxylin and eosin (HE) staining for the histological evaluation of GBM. After the MRI scan on day 14, the brains of the rats were harvested and fixed in 10% paraformaldehyde. The brain tissue was then embedded in paraffin wax and sectioned at a thickness of 5 μm. The sections were dewaxed in xylene and rehydrated by ethanol–water washes. After washing in water, the sections were incubated for 5 min with hematoxylin and rinsed in warm water for 10 min. Next, the sections were incubated for 3 min with eosin. Then, the sections were rehydrated using a series of ethanol–water washes and dewaxed in xylene. After 20 min, the slides were mounted. All the HE stains were observed using a fluorescence microscope (BZ-X810; KEYENCE CORPORATION, Osaka, Japan).

### 2.7. Statistical Analysis

The estimated parameter values are expressed as the means ± SDs. The between-group differences in the estimated parameter values, including the tumor volume, T_2_WI signal intensity ratio, MTR, ADC, FA, ICVF, ODI, ISO, and body weight, were analyzed by one-way ANOVA with Tukey’s multiple-comparison tests using Prism 9 (Version 9; GraphPad Software, San Diego, CA, USA). A *p*-value <0.05 was considered statistically significant.

## 3. Results

### 3.1. Effect of TMZ Treatment on Tumor Size

**Figure 3:**
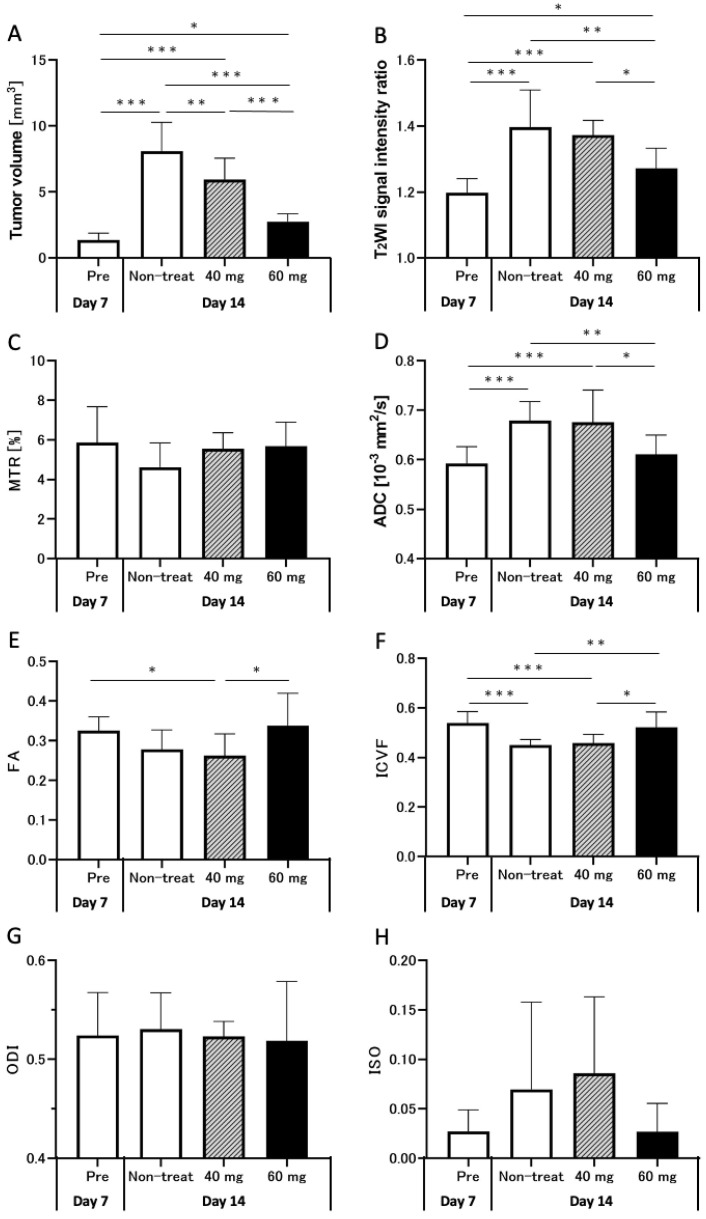
Graphs quantifying the tumor volume (**A**), T_2_WI signal intensity ratio (**B**), MTR (**C**), ADC (**D**), FA (**E**), ICVF (**F**), ODI (**G**), and ISO (**H**) in pre-treatment group on day 7 (white bars, *n* = 25) and non-treatment (white bars, *n* = 10), TMZ-40 mg (gray bars with diagonal lines, *n* = 7), and TMZ-60 mg (black bars, *n* = 8) groups on day 14 after transplantation. *: *p* < 0.05; **: *p* < 0.01; ***: *p* < 0.001; 40 mg, TMZ-40 mg; 60 mg, TMZ-60 mg; MTR, magnetization transfer ratio; ADC, apparent diffusion coefficient; FA, fractional anisotropy; ICVF, intracellular volume fraction; ODI, orientation dispersion index; ISO, isotropic volume fraction.

The tumor volumes and other MRI parameters on days 7 and 14 are shown in Figure 3. The tumor volumes on day 14 in the non-treatment (8.1 ± 2.1 mm^3^), TMZ-40 mg (6.0 ± 1.5 mm^3^), and TMZ-60 mg groups (2.7 ± 0.6 mm^3^) were significantly increased compared to those in the pre-treatment group on day 7 (1.4 ± 0.5 mm^3^). On day 14, the tumor volume in the TMZ-treatment groups was significantly lower than that in the non-treatment group (TMZ-40 mg: *p* < 0.01; TMZ-60 mg: *p* < 0.001). In addition, the tumor volume in the TMZ-60 mg group was significantly lower than that in the TMZ-40 mg group (*p* < 0.001).

### 3.2. Effects of TMZ Treatment on T_2_WI

**Figure 4:**
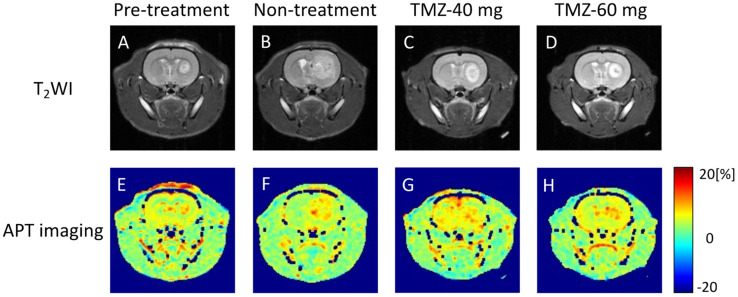
Representative T_2_W and APT images of pre-treatment group (**A**,**E**) on day 7 after transplantation and non-treatment group (**B**,**F**), TMZ-40 mg (**C**,**G**), and TMZ-60 mg (**D**,**H**) on day 14 after transplantation. TMZ, temozolomide; APT, amide proton transfer.

Representative T_2_WIs of rats in the pre-treatment, non-treatment, TMZ-40 mg, and TMZ-60 mg groups are shown in Figure 4A–D. In T_2_WI, the tumor tissue showed a higher signal intensity than normal tissue. The tumor size on day 14 tended to be smaller in the groups with higher TMZ doses. On day 14 post-injection, there was a heterogeneous signal within the tumor in the non-treatment and TMZ-treatment groups (Figure 4B–D).

The T_2_ signal intensity ratios on days 7 and 14 are shown in Figure 3B. The T_2_ signal intensity ratio in the non-treatment and TMZ-treatment groups was significantly increased compared to that in the pre-treatment group (non-treatment and TMZ-40 mg, *p* < 0.001; TMZ-60 mg, *p* < 0.05). On day 14, the T_2_ signal intensity ratio in the TMZ-60 mg group was significantly lower than that in the non-treatment (*p* < 0.01) and TMZ-40 mg groups (*p* < 0.05). The results confirmed that chemotherapy with 60 mg of TMZ suppressed the increase in T_2_ signal intensity ratio from day 7 to day 14.

### 3.3. Effects of TMZ Treatment on APT Imaging

Representative APT imaging of rats in the pre-treatment, non-treatment, TMZ-40 mg, and TMZ-60 mg groups is shown in Figure 4E–H. High MTR values were observed in the tumor marginal areas in all the groups on days 7 and 14. In the non-treatment group on day 14, the MTR low-signal area in the center of the tumor was increased compared to that in the pre-treatment group on day 7. In contrast, there was no increase in the MTR low-signal area in the center of the tumor on day 14 in the TMZ-treatment group compared to that on day 7 in the pre-treatment group.

The MTR values on days 7 and 14 are shown in Figure 3C. The MTR values in the non-treatment group on day 14 tended to decrease compared to the MTR values in the pre-treatment group on day 7 (non-treatment group: 4.6 ± 1.2% vs. pre-treatment group: 5.9 ± 1.8%). On day 14, there was no significant difference between the three groups, but the higher the TMZ dose, the smaller the decrease in MTR values from day 7 (TMZ-40 mg: 5.6 ± 0.7% vs. TMZ-60 mg: 5.7 ± 1.1%).

### 3.4. Effect of TMZ Treatment on DTI

**Figure 5:**
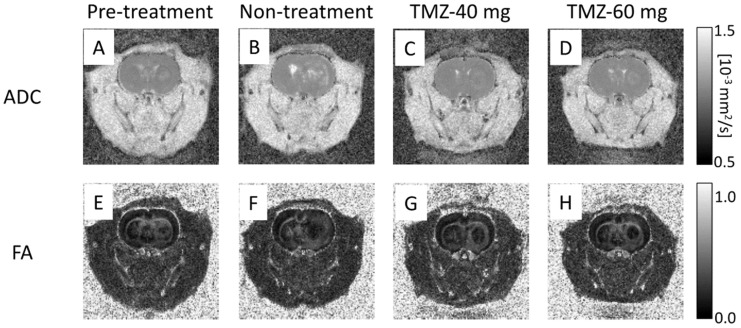
Representative ADC maps and FA maps of pre-treatment group (**A**,**E**) on day 7 after transplantation and non-treatment group (**B**,**F**), TMZ-40 mg (**C**,**G**), and TMZ-60 mg (**D**,**H**) on day 14 after transplantation. TMZ, temozolomide; ADC, apparent diffusion coefficient; FA, fractional anisotropy.

Representative ADC maps of rats in the pre-treatment, non-treatment, TMZ-40 mg, and TMZ-60 mg groups are shown in Figure 5A–D. The ADC map of the pre-treatment group on day 7 showed a slightly high signal intensity in the tumor area (Figure 5A), and the ADC map of the non-treatment group on day 14 showed a high signal intensity in the center and periphery of the tumor (Figure 5B). In the TMZ-treatment group, there was a slight contrast with normal tissue, showing a high signal intensity at the margin and a low signal intensity at the center (Figure 5C,D).

The ADC values on days 7 and 14 are shown in Figure 3D. The ADC values on day 14 in the non-treatment (0.68 ± 0.04 × 10^−3^ mm^2^/s) and TMZ-40 mg groups (0.68 ± 0.06 × 10^−3^ mm^2^/s) were significantly increased compared to those on day 7 in the pre-treatment group (0.59 ± 0.03 × 10^−3^ mm^2^/s) (*p* < 0.001). On the other hand, the ADC values on day 14 in the TMZ-60 mg group (0.61 ± 0.04 × 10^−3^ mm^2^/s) were not significantly different from those in the pre-treatment group on day 7. On day 14, the TMZ-60 mg group had significantly lower ADC values than the non-treatment (*p* < 0.01) and TMZ-40 mg groups (*p* < 0.05). Figure 3D shows that, the higher the dose of TMZ, the lower the increase in ADC from day 7 to day 14.

Representative FA maps of rats in the pre-treatment, non-treatment, TMZ-40 mg, and TMZ-60 mg groups are shown in Figure 5E–H. In all the groups, the tumors showed a uniform low signal intensity compared to normal tissue. The FA values on days 7 and 14 are shown in Figure 3E. The pre-treatment group on day 7 (0.33 ± 0.03) and TMZ-60 mg group on day 14 (0.34 ± 0.08) had a significantly higher FA value than the TMZ-40 mg group (0.26 ± 0.05) (*p* < 0.05). The graph shows that, the higher the amount of TMZ administered, the less the decrease in FA from day 7 to day 14.

### 3.5. Effect of TMZ Treatment on NODDI

**Figure 6:**
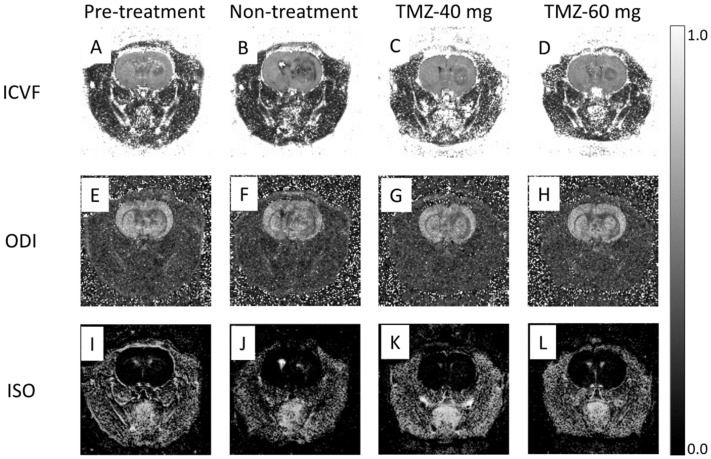
Representative ICVF maps, ODI maps, and ISO maps of pre-treatment group (**A**,**E**,**I**) on day 7 after transplantation and non-treatment group (**B**,**F**,**J**), TMZ-40 mg (**C**,**G**,**K**), and TMZ-60 mg (**D**,**H**,**L**) on day 14 after transplantation. TMZ, temozolomide; ICVF, intracellular volume fraction; ODI, orientation dispersion index; ISO, isotropic volume fraction.

Representative ICVF maps of rats in the pre-treatment, non-treatment, TMZ-40 mg, and TMZ-60 mg groups are shown in Figure 6A–D. In the pre-treatment group on day 7, the tumor showed a slightly lower signal intensity compared to normal tissue (Figure 6A). In the non-treatment group on day 14, a low signal intensity was observed in the center and periphery of the tumor (Figure 6B). In the TMZ-treatment group, the tumor margins showed a low signal intensity and the center of the tumor showed a signal intensity equal to that of normal tissue (Figure 6C,D). In addition, the non-treatment group on day 14 had a greater signal irregularity in the tumor than the TMZ-treatment group and the pre-treatment group.

The ICVF values on days 7 and 14 are shown in Figure 3F. The ICVF values on day 14 in the non-treatment (0.45 ± 0.02) and TMZ-40 mg groups (0.46 ± 0.03) were significantly decreased compared to those in the pre-treatment group on day 7 (0.54 ± 0.04) (both *p* < 0.001). However, ICVF values on day 14 in the TMZ-60 mg group (0.52 ± 0.06) were not significantly different from those in the pre-treatment group on day 7. On day 14, the TMZ-60 mg group had significantly higher ICVF values than the non-treatment (*p* < 0.01) and TMZ-40 mg groups (*p* < 0.05). The graph shows that, the higher the dose of TMZ, the smaller the decrease in ICVF from day 7 to day 14.

Representative ODI maps of rats in the pre-treatment, non-treatment, TMZ-40 mg, and TMZ-60 mg groups are shown in Figure 6E–H. In the pre-treatment group on day 7, the tumor showed a slightly lower signal intensity compared to normal tissue (Figure 6E). The ODI map of the non-treatment group on day 14 shows a low signal intensity in the central and peripheral areas of the tumor (Figure 6F). In the TMZ-treatment group on day 14, the tumor area showed a signal intensity equal to that of normal tissue, and the area around the tumor showed a low signal intensity (Figure 6G,H).

The ODI values on days 7 and 14 are shown in Figure 3G. There were no significant differences in the ODI values among the pre-treatment group on day 7 (0.52 ± 0.04), the non-treatment group (0.53 ± 0.03), the TMZ-40 mg group (0.52 ± 0.01), and the TMZ-60 mg group on day 14 (0.52 ± 0.06).

Representative ISO maps of rats in the pre-treatment, non-treatment, TMZ-40 mg, and TMZ-60 mg groups are shown in Figure 6I–L. In the day 7 pre-treatment group and the day 14 TMZ-60 mg group, there was no signal change due to the tumor (Figure 6I,L). In the day 14 non-treatment group, high-signal-intensity areas were observed in the center of the tumor (Figure 6J). In the TMZ-40 mg group, sparse high signal intensity was observed in the tumor area.

The ISO values on days 7 and 14 are shown in Figure 3H. The ISO values in the non-treatment (0.07 ± 0.08) and TMZ-40 mg groups on day 14 (0.086 ± 0.07) tended to increase compared to those in the pre-treatment group on day 7 (0.027 ± 0.02). On day 14, the ISO values in the TMZ-60 mg group (0.027 ± 0.03) tended to be lower than those in the non-treatment and TMZ-40 mg groups. In addition, the pre-treatment and the TMZ-60 mg groups showed less variation in ISO values than the non-treatment and TMZ-40 mg groups (pre-treatment: ±0.02; non-treatment: ±0.08; TMZ-40 mg: ±0.07; TMZ-60 mg: ±0.03).

### 3.6. HE Staining of GBM Model

**Figure 7:**
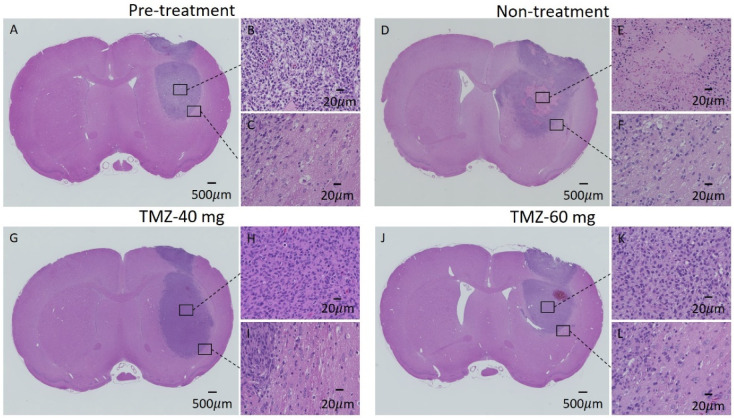
HE staining images of individuals in the pre-treatment group on day 7 and the non-treatment, TMZ-40 mg, and TMZ-60 mg groups on day 14. (**A,D,G,J**): Black bars equal 500 μm. (**B**,**C**,**E**,**F**,**H**,**I**,**K,L**): 20 μm. HE, hematoxylin and eosin.

**Figure 8:**
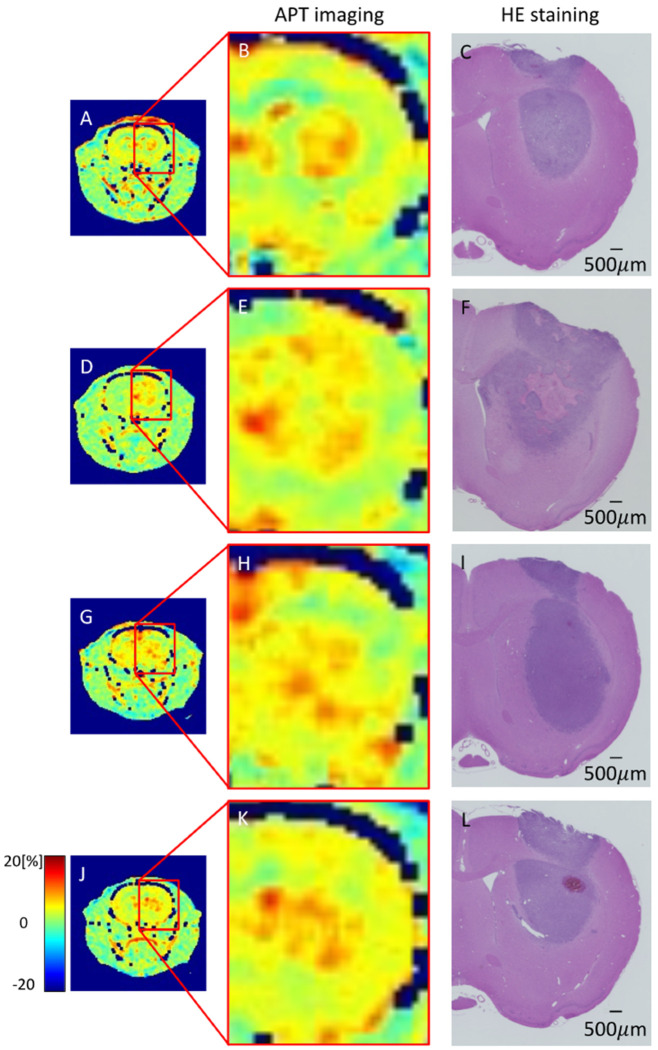
APT imaging and HE staining in pre-treatment group (**A**–**C**) on day 7 after transplantation and non-treatment (**D**–**F**), TMZ-40 mg (**G**–**I**), and TMZ-60 mg (**J**–**L**) groups on day 14 after transplantation.

Representative histological sections of tumors from rats in the pre-treatment, TMZ-40 mg, and TMZ-60 mg groups are shown in Figure 7A–L. No necrotic areas were observed in the pre-treatment group on day 7 and the TMZ-60 mg group on day 14 (Figure 7A,J). In contrast, necrotic areas were observed in all the individuals in the non-treatment group and in some individuals in the TMZ-40 mg group (Figure 7D,E). Figure 8 shows an APT image and an HE-stained image of rats in the pre-treatment group on day 7 and in the non-treatment, TMZ-40 mg, and TMZ-60 mg groups on day 14. The necrotic region in the center of the tumor observed by HE staining matched with the MTR low-signal-intensity region on APT imaging (Figure 8E,F).

### 3.7. Body Weight Changes in Rats Treated with TMZ

The average body weights of the brain tumor model rats on days 7 and 14 in the three groups are shown in Table 1. The mean weight of the non-treatment group increased from day 7 to 14, whereas the mean weight of the TMZ treatment group decreased from day 7 to 14. The ratio of the body weight at day 14 to the body weight at day 7 (day 14/day 7) was significantly lower in the TMZ-treatment group than in the control group (both *p* < 0.0001).

## 4. Discussion

In this study, TMZ chemotherapy was conducted with two different doses starting 7 days after transplantation in a rat GBM model. The multi-parameter values calculated from APT imaging and NODDI on days 7 and 14 after transplantation were used to evaluate the successive changes in each value due to tumor pathology. In the non-treatment group, necrotic areas were observed in the center of the tumor on day 14 after transplantation, which caused decreases in the MTR values of the APT imaging. In contrast, no necrotic areas were observed on day 14 after transplantation in the TMZ-60 mg group, and the MTR values did not decrease from days 7 to 14 after transplantation. This result may reflect the fact that TMZ treatment suppressed the formation of necrotic regions on day 14. It may be that the ICVF values calculated by NODDI could reflect the difference in the therapeutic effect on tumor cells between the TMZ-40 mg and TMZ-60 mg groups, as well as the ADC values calculated from DTI and the T_2_ signal intensity ratio calculated from T_2_WI. Therefore, these parameters may be able to distinguish the difference in tumor growth inhibition as a result of TMZ treatment.

In both the TMZ-treatment and non-treatment groups, significant increases in tumor volume were observed between days 7 and 14 after transplantation. It was confirmed that the TMZ treatment used in this experiment had an inhibitory effect on the growth of rat GBMs, but a small effect on tumor shrinkage. Previous studies have shown that rat glioma C6 cells are resistant to TMZ, the standard treatment for glioma [42]. The current results for TMZ treatment also show no reduction in tumor volume from pre-treatment compared to after 1 week of treatment. Therefore, we believe that the present results for tumor volume measurement show a similar trend to a previous study [42]. However, in a comparison between the three groups on day 14 after transplantation, the tumor volume in the TMZ-treatment groups was significantly smaller than that in the non-treatment group, and the tumor volume in the TMZ-60 mg group was significantly smaller than that in the TMZ-40 mg group. These results confirm that TMZ chemotherapy can inhibit the growth of GBMs as early as day 7 of treatment. In a previous study in rats, TMZ chemotherapy for glioma was reported to inhibit tumor growth and prolong life, and this trend was similar to that found in the present results [43,44]. The duration of TMZ treatment in this experiment was shorter than that for humans, and the TMZ dose was 20- to 30-fold higher per body weight than the TMZ dose used in clinical practice. Therefore, it is difficult to make a simple comparison between the TMZ-treatment method in this experiment and that in clinical practice. However, it is suggested that TMZ treatment may have had an early inhibitory effect on GBM.

On the other hand, the TMZ treatment in this study may have had significant side effects. In the current study, rats gained weight in the non-treated group from day 7 to day 14 after transplantation, whereas rats in the TMZ-treated group lost weight. Although TMZ treatment is the standard treatment for high-grade gliomas and is associated with relatively mild side effects [45,46], clinical studies have confirmed that increasing the TMZ dosage leads to stronger side effects and lower patient quality of life [47]. In the present study, it is suggested that the high dose per body weight may have resulted in stronger side effects, including the decrease in the body weight of the rats.

The T_2_ signal intensity ratio of the non-treatment group showed a significant increase from day 7 to day 14 after implantation. One possible reason for the increase in the signal intensity ratio is that the necrosis observed in the HE-stained sections on day 14 (Figure 7D) also increased the water content in the surrounding tumor area. APT imaging showed a relatively uniform signal distribution in the center of the tumor on day 7, but on day 14, a low-signal-intensity area of MTR was formed in the center of the tumor in the non-treatment group (Figure 4F). The MTR values of the non-treatment group on day 14 tended to be lower than those of the pre-treatment group on day 7 (Figure 3C). In the HE-stained section of the non-treatment group on day 14, a necrotic area with a very low cell density was observed in the center of the tumor, which coincided with the low-signal-intensity area on APT imaging.

Rapid growth of the tumor causes a lack of blood flow to the center of the tumor, leading to cell death and necrosis in that area. Necrotic areas with reduced tumor cell density may have reduced cytosolic protein content and mobile proteins, which are the source of the APT signal [32,33]. A previous study in rats reported that areas of both necrosis caused by radiotherapy and spontaneous necrosis seen in malignant gliomas show low MTR values [48,49,50,51]. In the necrotic area with reduced cell density, the MTR value calculated by APT imaging showed a low signal intensity, which is consistent with our results. On the other hand, in the TMZ-treatment group, although there was an increase in tumor size compared to day 7, no necrotic area in the center of the tumor was observed. Since no necrotic areas with low MTR values were observed (Figure 4G,H), it is suggested that the MTR values on day 14 in the TMZ-treatment group were similar to those on day 7 in the pre-treatment group without a decrease in the MTR values of the entire tumor. The present results, in which the treatment group had higher MTR values than the non-treatment group, show a different trend from previous studies, in which groups that were treated with molecular targeted therapy or radiotherapy showed lower MTR values [9,48,52,53]. This is probably due to the fact that the treatment used in this study only temporarily inhibited tumor growth, whereas the treatment used in the previous studies was able to reduce the growth potential of the tumor. In a previous study, the MTR value of GBM decreased at 4 to 6 weeks after the start of treatment [52]. In our TMZ treatment study, it may have been possible to observe a decrease in the MTR value after a longer period of treatment.

In the DTI and NODDI analyses, a high signal intensity for ADC and ISO and a low signal intensity for ICVF and ODI were observed in the necrotic region of the non-treatment group on day 14 compared to day 7 after transplantation. In a previous study, it was reported that high-grade gliomas showed a high ADC signal and a high ISO signal in the necrotic region [12,13,21], which is consistent with the signal change in the necrotic region in our experiment. The increase in ADC and ISO values suggests a decrease in cell density and an increase in the isotropic diffusion of water molecules in the necrotic region of the tumor center. The ICVF value, representing intracellular diffusion, is considered to have been reduced by the decrease in the number of tumor cells due to necrosis. Furthermore, the diffusion of extracellular water molecules is expected to shift from restricted diffusion to isotropic diffusion because there is less tissue to restrict extracellular diffusion in the necrotic region. Therefore, it is considered that the ODI value decreased and the ISO value increased in the necrotic region. Tumors were depicted as low-signal ICVF in all the groups on days 7 and 14 after transplantation. Previous studies have reported that gliomas cause a loss of axons and nerve fibers, detected as a decrease in ICVF [23,24]. It is suggested that there was a similar change in the current experiment.

Compared with the TMZ-40 mg group, the TMZ-60 mg group showed a greater effect on inhibiting tumor growth and a smaller signal change due to necrosis. In the quantitative evaluation, the higher-TMZ-dose group was closer to the values of the pre-treatment group on day 7 for the four diffusion parameters of the FA, ADC, ICVF, and ISO. The fact that TMZ treatment suppressed the changes in these parameters may reflect the tumor-growth-inhibitory effect of TMZ. This result may be similar to that in a previous study in which TMZ was administered to glioma model rats and the growth-inhibitory effect was confirmed by measuring the tumor volume and the use of histological staining, such as Ki-67 [44].

In the tumor periphery, changes in the calculated parameter values due to tumor growth were observed. The ADC map of the non-treatment group on day 14 showed a high signal intensity at the tumor periphery. In HE-stained cross-sections, many cavities were observed in the stroma at the tumor periphery of all the groups on days 7 and 14, which may have been due to water retention (Figure 7C,F,I,L). In the non-treatment group on day 14, the changes were more pronounced, with a larger number of cavities, which were widely observed around the tumor. Therefore, they are expected to have been detected as an increase in ADC. In a previous study, edematous areas were detected as increased ADC in the tumor periphery [11,12,13,54], which suggests that edematous areas were formed in the tumor periphery in the non-treatment group on day 14. Low-signal ICVF areas (Figure 6B) and low-signal ODI areas (Figure 6E–H) were also observed in the tumor periphery. In a previous study, the area around the glioma showed a low-signal ICVF region due to edema [20,24]. Based on this report, the low-signal ICVF region at the tumor periphery observed in the present experiment is expected to be due to edema. The decrease in ICVF values due to edema may be due to the relative decrease in neurite density caused by water retention in the stroma. Furthermore, the low-signal ICVF region at the tumor periphery was consistent with the high-signal ADC region due to edema observed in the non-treatment group on day 14.

In the ODI changes in the tumor periphery, previous studies have demonstrated that tumor invasion and edema lead to an increased ODI and increased extracellular diffusion, respectively, and that decreased extracellular space also affects the ODI [21,22]. Based on these reports, the decrease in ODI values around the tumor in this experiment is not likely to have been due to tumor invasion and edema. The orthotopic model in this experiment showed extremely fast growth compared to GBM that develops in humans. It is considered that, as the tumor grows, the surrounding tissues are compressed and the scattering of neurites decreases. The present results suggest that the effect of compression due to tumor growth was greater than the effect of infiltration and edema. The decrease in neurite orientation dispersion due to compression may have been reflected in the decrease in ODI.

There were several limitations to our study. First, GBMs in orthotopic transplantation models grow faster than GBMs observed in clinical practice, and this allows the observation of GBMs for about 2–3 weeks after transplantation, but it is difficult to observe them for longer periods of time. Although the orthotopic transplantation model of GBM is a highly reproducible experimental model, the use of transplanted cancer cells results in rapid growth and a high risk of death for the model animals. In order to evaluate the longer-term pathogenesis of tumors, we could adjust the number of cells to be transplanted or consider using slow-growing models such as ethyl-nitrosourea-induced brain tumor models [10]. Second, the dosage, method of administration, and duration of TMZ treatment for GBM need to be investigated further. Chemotherapy with TMZ alone, as used in this experiment, was less effective in inhibiting tumor growth. We would like to observe the changes in each parameter when TMZ treatment is combined with radiation therapy or molecular targeted therapy to achieve therapeutic effects such as a reduction in tumor volume and MTR values. Moreover, in future studies, we would like to consider administering treatment prior to day 7. Finally, the addition of an MRI point would be useful. In this experiment, MRI was performed at two points, day 7 and day 14 after tumor implantation. We believe that adding multiple measurement points before day 7 and after day 14 should be considered to evaluate the changes in MTR values at the time when cells take hold and begin to proliferate, and the proliferative potential of tumors after TMZ treatment. In addition, we would like to consider changing the position of the ROI for measurement. For example, there is a method where ROIs can be placed in areas with and without contrast-enhanced effects in gadolinium-based contrast-enhanced MRI. In a previous study, detailed histological examinations of rat brain samples with different staining methods (hematoxylin and eosin, glial fibrillary acid protein (GFAP), von Willebrand factor (vWF), or luxol fast blue (LFB)) were performed [55]. They show that the in vivo MRI evaluation of myelinated or demyelinated regions of a tumor is beneficial for a comprehensive understanding of the mechanisms involved in brain tumor development and tumor cell migration. In addition, the APT signal of a glioma tumor is prominently influenced by the protein concentration and tumor pH. Therefore, it will be useful to use some biomarker staining agents such as Neuro-2A, Iba1, and GFAP for neurite and glial cell changes for the evaluation of tumor characteristics in future experiments [27].

## 5. Conclusions

The multi-parameter values calculated from APT imaging and NODDI for GBM were able to highlight the differences in intratumor structure in TMZ-treatment and non-treatment groups. Among the calculated parameters, the ICVF of the NODDI may be useful as a parameter for determining the effect of tumor growth inhibition, as well as T_2_ signal intensity ratio and ADC.

## Figures and Tables

**Table 1:** Weight changes due to side effects of TMZ treatment. Values are expressed as means ± standard deviations (SDs). ns: not significant.

Average Body Weight (g)			
Group	Non-treatment	TMZ-40 mg	TMZ-60 mg
Day 7	198.2 ± 14.9	194.1 ± 12.1	211.9 ± 7.4
Day 14	224.1 ± 13.0	189.7 ± 16.4	204.3 ± 10.4
Average body weight ratio			
Day 14/Day 7	1.13	0.98	0.96
**Compared groups**		***p***	
Non-treatment	TMZ-40 mg	<0.0001	
Non-treatment	TMZ-60 mg	<0.0001	
TMZ-40 mg	TMZ-60 mg	ns	

ns: not significant.

## Data Availability

The data presented in this study are available on request from the corresponding author.
